# Eagerness for Extensive Practice

**Published:** 1854-03

**Authors:** 


### *Eagerness for an Extensive Practice.*

There is very naturally an active strife among medical men to obtain business. The great ambition is to be known as the possessor of a large practice, rather than as a writer, a teacher, or a wise or learned man. There are indeed striking exceptions to this remark, as some excellent authors are found in our ranks. But the great majority pursue a different path; and some of them, as it would sometimes appear, would be glad to monopolize all the sick if they could. The instances of disagreement, ill will, and unneighborly conduct between gentlemen in our profession, often proceed from a real or imagined trenching upon each other's beat of practice, than from any other cause. We know persons of generous natures, kind, charitable, and obliging in every other respect; yet, if another physician happens, through the caprice of a patient, or from any circumstance, to attend on one of their customers, they give way to a paroxysm of rage. The old-fashioned rounds of family practice are gradually becoming smaller, even in the country towns. A prodigious increase in physicians, beyond the actual demand, together with the multiplication of irregular pretenders, conduces to this result. But this is no cause of complaint, and it is quite useless to fritter life away in a pet, because we cannot retain things in the condition in which we found them. Instead of quarrelling with the world because it will not conform to our convenience, it is far better to shape ourselves to the times. A happy disposition is worth more than a vast estate. The public avoid those who exhibit too much eagerness in the pursuit of professional patronage. They distrust those who complain of being neglected. No talent, accompanied by industry and a good moral character, goes unrewarded in this country. People cannot be dragooned into employing certain medical advisers.

When avarice gets the ascendancy, physic becomes a mere instrumentality for rapid accumulatlon. Science is invariably neglected when a morbid craving for trade takes possession of the man. Success in any calling depends upon economizing hours, and improving opportunities for advancement. But even when all has been done, it is ridiculous to lament because our patrons are fickle and our friends die off and give place to strangers. Philosophy and common sense are studies worth pursuing by those of our brethren who are liable to be unduly effected by these changes. But above all, they should avoid giving way to envious repining. As a general thing, the difficulty is in ourselves, when our services are not as extensively required as we think they ought to be. The truest test of adequate preparation for the details of practice, is the constant and continued demand for the exercise of our knowledge and skill.—*Boston Medical and Surgical Journal.*

